# Current insights on predicting vestibular diseases using machine learning

**DOI:** 10.55730/1300-0144.6062

**Published:** 2025-07-14

**Authors:** Emre SÖYLEMEZ, Muhammed Mustafa ŞEKER

**Affiliations:** 1 Department of Audiometry, Vocational School of Health Services, Karabük University, Karabük, Turkiye; 2 Department of Audiology and Speech Pathology, Institute of Health Sciences, Ankara University, Ankara, Turkiye

**Keywords:** Machine learning, vestibular diseases, BPPV, Ménière’s disease, vestibular neuritis

## Abstract

The vestibular system is one of the three main systems responsible for maintaining balance and posture. Accurate vestibular inputs enable the perception of the head’s position and movement in space, and ensure coordination between head movements, eye movements, balance, and posture. Any dysfunction in the peripheral vestibular end organs, the vestibular nerve, or the central vestibular system may lead to vertigo, dizziness, and gait disturbances in individuals. Some syndromes that cause vertigo symptoms can be life threatening. Although peripheral vestibular pathologies are generally benign, they can reduce patients’ quality of life, cause falls, and hinder independence. Therefore, the diagnosis and management of vestibular disorders are of great importance. However, due to the complex structure of the vestibular system and the complexity of its symptoms, some vestibular diseases may go undiagnosed or be misdiagnosed. Machine learning (ML), a subfield of artificial intelligence, enables computer systems to learn patterns and relationships from data and make predictions or decisions. The growing capabilities of ML in data processing combined with the needs of healthcare, offer significant opportunities in early diagnosis of diseases, treatment planning, and personalization of healthcare services. The present review provides a general overview of the prediction of vestibular disorders using ML.

## 1. Introduction

The vestibular system is one of the three main systems responsible for maintaining balance, along with the proprioceptive and visual systems [1]. The vestibular end organs located in the inner ear (3 semicircular canals and 2 otolith organs) are stimulated during head movements, and this information is transmitted to the central system [1]. This is how the position and movement of the head in space are perceived. When the vestibular system functions properly, coordination between head movements, eye movements, balance, and posture is maintained. Any dysfunction in the peripheral vestibular end organs, the vestibular nerve, or the central vestibular system may lead to vertigo, dizziness, and gait disturbances in individuals. Therefore, the diagnosis and treatment of vestibular disorders are crucial for preventing falls and ensuring independence.

The vestibular system is a complex structure and network of neural pathways that, together with many other systems, serves a wide variety of functions [1]. In recent years, the approach to vestibular disorders has significantly evolved. During this period, new guidelines related to vestibular disorders and studies including treatment approaches for these conditions have been published [2]. However, despite all these advances, the underlying cause of vestibular pathology cannot be fully understood in some cases. One study reported that 45% of patients with dizziness remained undiagnosed, while 18% were misdiagnosed [3].

Machine learning (ML) is a subfield of artificial intelligence (AI). ML is defined as the ability of a machine to mimic intelligent human behavior [4]. The foundations of ML date back to the 1950s [5]. Since then, ML has been used in multiple areas, especially in recent years. According to one report, approximately 86% of healthcare institutions use some form of ML system, and more than 80% of healthcare leaders have an AI plan [4].

The excessive growth and complexity of data have increased the risk of human errors in processes. The growing capability of ML to process data combined with human needs, presents an opportunity to reduce these risks. Today, ML has acquired advanced decision-making, problem-solving, and computational abilities. As a result, it has been applied in many fields [6]. ML is widely used across various medical disciplines, including heart failure management, clinical decision support in clinical medicine, and medical imaging [4].

In the development of ML models, data is typically divided into 2 groups to ensure reliable learning, representative populations, and unbiased predictions: training and testing datasets. In the training dataset, the characteristic features of the attributes associated with correct and incorrect outcomes are identified and processed according to the specifications of the algorithm. In the training phase, the dataset is introduced to the algorithm and learning takes place. The trained algorithm is then evaluated using the testing dataset that consists of similar but previously unseen data, to determine accuracy and performance of the model. This process objectively assessess the capabilities of the model, free from bias [7]. Once an algorithm successfully completes both the training and testing phases with acceptable results, it can be implemented in healthcare environments [5].

## 2. Machine learning

ML models are divided into 3 types, depending on the problem they are trying to solve: supervised learning, unsupervised learning, and reinforcement learning (RL) [4].

### 2.1. Supervised learning

Supervised learning models are used in situations where the desired outcome is predefined and the data is explicitly labeled according to that outcome [8]. In these models, each data instance consists of an input (features) and a corresponding output (label). For example, the output variable may represent the presence or absence of a condition such as diabetes or hypertension [4]. Through such labeled data, the model learns the relationship between inputs and outputs, enabling it to make predictions in similar future scenarios. The supervised learning process begins with the formulation of a clear problem statement related to the issue being addressed. Subsequently, relevant and meaningful data is collected and prepared to solve this problem [9]. Typically, the collected data undergoes 4 essential stages: classification, preprocessing, model training, and testing [9,10]. These stages are critical for enhancing the accuracy and generalizability of the model. Supervised ML is widely applied in medicine and health sciences. It is used for disease diagnosis, risk analysis, prediction of treatment outcomes, and optimization of healthcare services. The frequent availability of labeled data in such domains provides an ideal environment for supervised learning models to generate effective and reliable results.

### 2.2. Unsupervised learning

In unsupervised learning, there is no predefined target variable, and the data is unlabeled. These types of algorithms are used to uncover hidden patterns, relationships, or structures within data. The model operates solely based on inputs and must infer the underlying nature of the data to interpret the results. In other words, without relying on any predefined outputs, the algorithm aims to identify meaningful structures by analyzing similarities or differences among data points [11].

Among unsupervised learning methods, clustering holds significant importance [12]. Clustering involves grouping data with similar characteristics into clusters based on specific criteria. This process is particularly effective for discovering meaningful structures in large datasets. However, the results obtained through clustering algorithms must be carefully evaluated. These algorithms may exaggerate similarities between data points, potentially causing distinctly different data to be grouped together. Such misgroupings can lead to overgeneralization and may result in incorrect decision-making.

Unsupervised ML techniques are not limited to clustering. Other commonly used methods include dimensionality reduction, feature extraction, and anomaly detection. In particular, anomaly detection is effective in identifying unusual patterns in datasets and is frequently used in scenarios such as fraud detection, system error identification, or the detection of rare events [4]. In this context, unsupervised learning methods have the potential to provide valuable insights across a wide range of fields, including healthcare, finance, security, and marketing.

### 2.3. Reinforcement learning

RL serves as a practical approach particularly focused on decision-making processes. In this learning paradigm, the system interacts with its environment by performing actions to achieve specific goals, receiving feedback in the form of rewards or penalties based on these actions. The model updates its future decisions according to the feedback received, and this process occurs through trial and error. Over time, the system learns which actions yield more favorable outcomes and utilizes this knowledge to enhance its performance. This cycle continues until the system produces the desired output; each new action and decision is shaped by the experience gained from previous feedback [13].

Decision-making in healthcare is rarely linear and often requires consideration of numerous variables, uncertainties, and interactions. In this context, RL offers potential for designing decision support systems that assist clinicians in selecting treatment options or intervention strategies. RL models can be particularly effective in complex processes such as developing personalized treatment plans, monitoring dynamic treatment responses, and achieving optimal health outcomes [14]. However, the application of RL in healthcare also presents several challenges. For instance, evaluating the accuracy and efficacy of system decisions, defining an appropriate reward function, and establishing a robust feedback mechanism are critical components that must be carefully addressed.

## 3. Machine learning in health sciences

The use of ML in the healthcare sector is becoming increasingly widespread. Research has shown that ML can offer significant benefits in enhancing the quality of healthcare services, supporting disease management, enabling early diagnosis, and reducing hospital operational costs [15]. In brief, ML has the potential to contribute to the improvement and more efficient delivery of healthcare services. In studies conducted within the fields of health sciences and medicine, supervised learning models are most commonly utilized, including algorithms such as naive Bayes, random forest (RF), support vector machines (SVM), decision trees, logistic regression, k-nearest neighbors (KNN), linear discriminant analysis (LDA), artificial neural networks (ANNs), multilayer perceptron (MLP), and radial basis function kernel methods.

Recent studies show that ML has been widely used for a variety of applications, including the diagnosis of COVID-19, identification of heart disease and optimal diagnostic algorithms, prediction of diabetes and diabetes-related risks, classification of liver diseases, early detection of various cancers, adjustment of drug dosages, prediction of heart attack risk, identification of thyroid diseases, early prediction of Alzheimer’s disease, and early diagnosis of kidney diseases [15]. With the growth of large-scale data, particularly through the storage of health information in systems such as electronic medical records, the use of ML in healthcare is expected to expand even further.

### 3.1. Commonly used algorithms in health sciences

#### 3.1.1. Support vector machines

SVM were introduced in 1979 [16]. This algorithm seeks a separating hyperplane that best distinguishes between input data. The primary goal of SVM is to achieve an accurate separation while maximizing the distance between the hyperplane and the data points that lie closest to it but on opposite sides of the decision boundary. These closest data points are known as support vectors.

To handle nonlinear separations, SVM can be extended through the use of kernel functions that enable the construction of nonlinear separating hyperplanes. Since only the support vectors are required to define and store the trained model, SVM is highly memory efficient. It is primarily used in classification and regression problems [17].

#### 3.1.2. Naive Bayes

Naive Bayes is a multiclass algorithm based on the assumption of independence among features. The term “naive” refers to this assumption of independence [18]. This implies that there are no relationships among the feature variables. In other words, each feature in the data is considered independent of the others.

This assumption of independence simplifies the calculations of the algorithm and reduces model complexity. It enables fast and efficient computation, particularly in problems involving a large number of features. However, this assumption does not always hold true for real-world data [19], and therefore, the algorithm has certain limitations. First, some features in real datasets may be correlated, and this should be considered when evaluating the results produced by the algorithm. Second, due to its reliance on the independence assumption, the algorithm may not always achieve high predictive performance. Despite these limitations, when applied with appropriate consideration, the naive Bayes algorithm can yield successful results in many classification tasks [12].

#### 3.1.3. Decision trees

Decision trees are classification and regression algorithms based on a tree structure composed of a root, nodes, and leaves [20]. In this structure, dependent and independent variables are represented using a series of test questions and conditions, forming a hierarchical tree. Starting from the root node, the tree splits the data according to feature values and ultimately assigns prediction values at the leaf nodes. Decision trees can be used to classify or predict data in a simple and interpretable manner [20].

The primary goal of decision tree algorithms is to develop a model capable of predicting the value of a target variable by learning rules derived from the input features. These models can be visualized, making it easier to explain and trace the decision-making process. While decision tree algorithms are widely used in classification problems, they are also capable of processing both categorical and continuous data. However, when the dataset is small or the tree contains too many branches and leaves, classification errors may increase. Furthermore, decision trees are prone to overfitting. To mitigate this issue, techniques such as pruning are used to limit the complexity of the tree and prevent overfitting [12].

#### 3.1.4. K-nearest neighbor

The KNN algorithm is a relatively simple ML approach used to solve classification and regression problems. When a new data point is introduced, the algorithm identifies the *k* nearest data points in the training set and predicts the class of the unknown data point based on the class labels of these neighbors. Various distance metrics can be used for this purpose; however, Euclidean distance is most commonly used [21]. Advantages of KNN include ease of implementation, flexibility in selecting different features and distance metrics, and the ability to naturally handle multiclass problems. Moreover, it can yield accurate results when applied to large and representative training datasets. Nonetheless, the algorithm also has certain drawbacks. Most notably, KNN is considered a lazy algorithm, meaning classification is only performed when a prediction for an unknown data point is required. This can lead to high computational costs, as extensive searches over the training data are necessary for each new classification. Additionally, selecting an appropriate distance function is critical, as an unsuitable choice may negatively impact the accuracy of the algorithm [22].

#### 3.1.5. Logistic regression

Logistic regression is an algorithm utilized in both classification and regression analyses. This method examines the relationship between independent and dependent variables, assisting in determining whether a given variable should be included in the model and assessing its importance. The algorithm begins with randomly initialized parameters and continuously seeks to minimize the error rate using a defined loss function. One of the major advantages of logistic regression is its ability to provide predictions in the form of interpretable probabilities. However, this model may be inadequate in addressing nonlinear relationships [23].

#### 3.1.6. Random forest

RF aims to eliminate the overfitting tendency observed in decision tree algorithms. This algorithm constructs a large number of decision trees by randomly selecting subsets of the dataset and features, subsequently combining them into a forest structure. This approach results in the creation of multiple diverse trees that are not pruned, thereby helping to mitigate overfitting issues [24]. Consequently, RF stands out as an ML algorithm that delivers high performance and reliable outcomes. However, its primary disadvantage is that training an RF typically takes longer than training individual decision trees.

#### 3.1.7. ADABoost

ADABoost is one of the ensemble learning methods that aims to construct a strong predictive model by combining multiple weak learners that are typically simple models such as decision trees. The algorithm adopts a weighted approach to minimize the classification errors of each learner, thereby iteratively improving overall model performance. Effectively applied to both classification and regression tasks, ADABoost generates superior results in terms of accuracy and generalization performance [25].

#### 3.1.8. Artificial neural networks

Inspired by the human brain, ANNs are capable of successfully modeling both linear and nonlinear functions. An ANN consists of computational units known as artificial neurons that are organized into layers [26]. Each layer processes inputs and transmits the results to subsequent layers. Typically, an ANN includes 3 layers. The input layer receives and presents data. The hidden layer is where data are processed and patterns are learned. The output layer provides final predictions or results [27]. Each artificial neuron receives multiple inputs, multiplies them by assigned weights, and sends the weighted sum to an activation function. The output of this function determines the response of the neuron that is then transmitted to other neurons. For effective modeling, a sufficient number of layers must be used to capture the relationship between any given input *x* and output *y*. There is no strict limitation on the number of hidden layers in ANNs; the number can be adjusted based on the nature of the data. However, increasing the number of layers also increases the complexity of the network and prolongs the training time [26]. Training an ANN involves adjusting the weights of the neurons to approximate the actual outcomes as closely as possible.

## 4. Machine learning in vestibular diseases

Dizziness is a symptom that affects approximately 20% of the general population [28]. Determining its underlying cause is complex due to the wide variety of associated conditions [29]. The pathology may originate from either central or peripheral disorders. Peripheral vestibular disorders are among the most common etiologies of dizziness/vertigo. The most prevalent peripheral vestibular disorders include benign paroxysmal positional vertigo (BPPV), vestibular neuritis (VN), and Ménière’s disease (MD). The diagnosis of these conditions is primarily based on patient history, vertigo/nystagmus characteristics, and various clinical tests. Utilizing these data in ML may assist in medical decision-making for vestibular disorders [29].

## 5. Methods

This review was conducted to examine the use of ML approaches in the prediction and diagnosis of vestibular disorders. Although it was not designed as a full systematic review, the literature selection process was structured and methodologically guided. A comprehensive search was carried out using the PubMed and Google Scholar databases to identify relevant studies published between January 2010 and December 2024. The search was performed using combinations of the following keywords: “machine learning,” “vestibular disorders,” “dizziness,” “vertigo,” “BPPV,” “Meniere’s disease,” “vestibular neuritis,” “vestibular neuropathy,” “vestibular hypofunction,” and “artificial intelligence.”

Studies were included based on the following criteria:

- Focus on the modeling, validation, or clinical application of ML algorithms in the context of vestibular disorders.
- Reporting the use of ML in vestibular assessment via mobile health tools or telemedicine platforms.

Exclusion criteria were as follows:

- Studies not directly related to ML or vestibular dysfunctions.
- Review articles without original analysis or technical content.
- Studies for which the full text was not accessible.

A total of 45 articles were initially identified. After screening according to the inclusion and exclusion criteria, 15 studies were included in the review. These studies are discussed under 6 headings.

### 5.1. Benign paroxysmal positional vertigo

BPPV is one of the most common peripheral vestibular disorders, accounting for approximately one-quarter of clinical vertigo cases. Its lifetime prevalence is estimated at 2.4%, with a recurrence rate of 50% [30]. The vestibular system detects linear and angular acceleration of the head during movement, playing a critical role in maintaining balance by stabilizing gaze, head, and trunk position. In BPPV, due to dysfunction of the vestibular system, patients often suffer from severe episodes of vertigo [31,32].

BPPV is believed to result from the displacement of otoconia from the utricle into the semicircular canals (SCC), where these dislodged particles trigger abnormal vestibular stimuli [33]. As a consequence, patients experience brief but intense vertigo attacks, typically lasting for seconds and triggered by changes in head position [34]. Diagnosis is primarily based on characteristic nystagmus observed during provocative maneuvers. However, in certain cases, fatigue or oversight by the clinician may lead to missed diagnoses. One study reported the sensitivity and specificity of the Dix-Hallpike maneuver, a commonly used diagnostic test, as 79% and 75%, respectively [35]. Following accurate diagnosis, BPPV can often be effectively treated through canalith repositioning maneuvers.

In the literature, ML approaches aimed at detecting BPPV have typically utilized features such as characteristics of nystagmus, patient history, and vertigo-related data. Wu et al. [36] analyzed the characteristics of nystagmus observed during provocative maneuvers using a combination of one-dimensional (1D) models and deep learning algorithms to predict BPPV. In their study, the overall model performance was reported with micro and macro level AUC-ROC values of 0.982 and 0.965, respectively. The authors further noted that the developed hybrid algorithm achieved the highest performance in predicting right and left posterior canal BPPV (PC-BPPV), with corresponding AUC-ROC values of 0.991 and 0.979, respectively. Similarly, Lu et al. [30] analyzed patients’ eye movement videos using a multimodal deep learning model and reported an accuracy of 81.7% in diagnosing BPPV. Eye movement and nystagmus analysis provide valuable objective markers that can distinguish between various BPPV subtypes such as posterior, horizontal, and anterior canal involvement. However, this method is generally restricted to specialized clinical settings because it requires specific equipment and expert interpretation, making its use challenging in remote or resource-limited environments.

Gait is a delicate task, and symptoms caused by BPPV negatively affect individuals’ functionality and walking ability. Zhang et al. [37] evaluated the gait performance of BPPV patients and healthy controls using an accelerometer. They aimed to use this data in ML to predict both the presence and severity of BPPV. The authors reported that an SVM model based on gait variables distinguished BPPV patients from healthy controls with 78% accuracy. Additionally, the model classified the disabling effects of dizziness caused by BPPV according to dizziness handicap inventory (DHI) scores as mild, moderate, and severe with average accuracies of 0.83, 0.85, and 0.96, respectively. Similarly, Hu et al. [38] evaluated the gait patterns of individuals with BPPV and vestibular migraine (VM) using an accelerometer. The authors noted that both VM and BPPV patients had more cautious gait patterns compared to healthy individuals. Using these parameters, the researchers developed 10 different ML models to distinguish between these conditions. The study concluded that impaired gait stability features enabled differentiation between BPPV and VM with an AUC of 0.854 and an accuracy of 83.9% using the RF algorithm [38].

The DHI is a widely used psychometric assessment tool designed to determine the severity of emotional, functional, and physical disability caused by dizziness [39]. Masankaran et al. [40] aimed to distinguish between PC-BPPV and horizontal canal (HC)-BPPV using DHI scores in ML. They trained 4 different algorithms (RF, SVM, KNN, and naive Bayes) with statistically significant DHI scores. The authors reported that the naive Bayes model achieved the highest accuracy, reaching 73.91%.

Demographic characteristics and medical history provide valuable information for the prediction of BPPV. A thorough and systematic clinical assessment that includes detailed symptom chronology, identification of positional triggers, and a history of prior episodes can substantially improve diagnostic accuracy and help differentiate BPPV from other vestibular disorders such as VM, MD, and central vertigo. Khani et al. [41] utilized demographic and clinical data from 7760 patients to develop ML models for predicting the presence of BPPV. The researchers reported that the gradient boosting model predicted BPPV with an accuracy of 85.42%. Patient medical history and symptom characteristics, while less specific for differentiating BPPV subtypes, are more accessible and feasibile. These data can be collected remotely through telemedicine platforms, enabling preliminary screening and monitoring without specialized hardware. Nevertheless, reliance on subjective reports may reduce diagnostic precision compared to objective measurements.

Differences in algorithm performance across studies are mainly due to variations in data type, quality, and size. Complex models, like deep learning, excel with large, high-dimensional datasets, while simpler algorithms may perform better with smaller, structured data. Additionally, factors such as parameter tuning and evaluation methods affect results. Therefore, the best algorithm depends on the specific dataset and clinical context.

### 5.2. Ménière’s disease

MD is an inner ear disorder characterized by hearing loss, tinnitus, and vertigo [42]. In most cases, the disease progresses slowly and significantly impairs the affected individual’s social functioning. The pathogenesis of MD remains unclear; however, the primary pathohistological feature is endolymphatic hydrops [43]. Although the exact etiology of the disease is still uncertain, various theories suggest that genetic and environmental factors play a major role [44]. In the diagnosis of MD, medical history, radiological imaging, and audio-vestibular tests are of critical importance [45].

Liu et al. [46] used air conduction thresholds from pure-tone audiometry tests to predict MD. The authors trained 5 classical ML models using this data: logistic regression, SVM, decision trees, RF, and light gradient boosting (LGB). They reported that the LGB model achieved the best performance, with an accuracy of 87%, sensitivity of 83%, specificity of 90%, and an AUC-ROC curve of 0.95.

Shew et al. [47] utilized perilymphatic microRNA (miRNA) profiles in various ML models to detect MD. Perilymph samples were collected during labyrinthectomy (MD, n = 5), stapedotomy (otosclerosis, n = 5), and cochlear implantation (sensorineural hearing loss [SNHL], n = 9). The authors noted that 2 of their ML models distinguished MD from conductive hearing loss with 100% accuracy. However, the best-performing ML model achieved a success rate of only 66% in differentiating MD from SNHL. The main limitation of Shew et al. [47] was the small sample size. This limited data reduces the statistical power of the ML models and increases the risk of overfitting. Additionally, the relatively low performance (66%) in distinguishing MD from sensorineural hearing loss (SNHL) suggests that the model may have difficulty differentiating diseases with overlapping sensorineural features.

Wang et al. [48] applied ML to distinguish between 114 patients with MD and 160 with VM by using medical histories and audio-vestibular test findings. The authors developed 3 models using 10 different ML algorithms. Model 1 included all available data (medical history, acute videonystagmography, video head impulse test, vestibular-evoked myogenic potentials, caloric testing, and audiogram). Model 2 utilized history, audiogram, and caloric test data. Model 3 used only history. These models achieved accuracies of 97.81%, 94.53%, and 92.34%, respectively, in differentiating MD from VM. The authors concluded that ML could accurately distinguish VM from MD and noted that model 3 could be implemented in primary healthcare settings for differential diagnosis. The authors’ development of multiple ML models using different data combinations allowed for comparison of how ML algorithms perform across various clinical datasets and showed their potential for practical application.

Nystagmus characteristics are considered less reliable in diagnosing MD compared to BPPV, primarily because the direction and pattern of nystagmus can vary significantly across different stages of the disease [49]. This variability reduces the consistency of nystagmus-based markers for MD classification. Consequently, clinical history and audiovestibular test findings are more likely to serve as useful inputs for ML models targeting MD diagnosis.

### 5.3. Vestibular neuritis

Vestibular neuritis (VN) is characterized by acute spontaneous vertigo lasting for several days without any auditory symptoms. VN is the third most common peripheral vestibular disorder, following BPPV and MD [50]. Although the exact etiology of VN remains unclear, it is believed that viral inflammation of the vestibular nerve ganglion plays a central role [51]. VN, along with posterior circulation stroke (PCS), is classified as an acute vestibular syndrome (AVS) that represents one of the most frequent reasons for emergency department visits [51]. Differentiating PCS from VN is critical since PCS is potentially life threatening, whereas VN is relatively benign and self limiting [52].

Wang et al. [52] utilized video head impulse test (vHIT) data from 301 patients to distinguish PCS from VN using 4 different ML models, with the best-performing algorithm achieving an accuracy of 87.8%. The strength of this study lies in the relatively large sample size and the direct comparison of multiple algorithms, allowing identification of the most suitable model for this diagnostic task. Similarly, Korda et al. [53] applied a neural network model to vHIT data from 57 emergency department patients presenting with AVS and achieved an accuracy of 87.9% in differentiating acute unilateral vestibulopathy (VN) from stroke. These findings suggest that ML can achieve performance comparable to conventional diagnostic thresholds and enable automated analysis. However, the relatively small sample size raises concerns about potential overfitting and limits the generalizability of the results.

### 5.4. Other vestibular diseases

The primary goal in vestibular disorders is to distinguish between peripheral and central pathologies. Subsequent objectives include diagnosing the specific disorder and implementing appropriate treatment. Anh et al. [29] applied balance tests from 497 patients with peripheral vestibular pathology (PVP) and 512 non-PVP patients to 5 different ML models. The authors reported that the best performance was achieved by the SVM model, with overall model accuracies ranging between 76% and 79%. Tsai et al. [54] utilized inertial measurement units (IMUs) to administer the Romberg test and investigated whether IMU parameters could be used in ML to objectively detect vestibular hypofunction (VH). The authors reported that the RF model achieved 94% accuracy, while the SVM model distinguished VH from healthy individuals with 97% accuracy. Similarly, Pathirana et al. [55] aimed to detect semicircular canal (SCC) abnormalities by using vHIT parameters in ML. They found that the linear SVM model identified VH with 100% accuracy. Overall, the study concluded that ML could assist clinicians in making more efficient diagnoses of vertigo.

These studies highlight the potential of ML to support clinicians in the diagnosis of vestibular disorders with promising accuracy rates. However, differences in sample sizes, feature selection, and model types across studies may influence performance outcomes. While most research shows encouraging results, further work focusing on improving model interpretability, addressing possible biases, and validating findings on larger and independent datasets will be valuable to strengthen the clinical utility and generalizability of these approaches.

### 5.5. Detecting fall risk with machine learning

Imbalance and falls are among the most significant clinical issues faced by elderly individuals. Falls are a leading cause of mortality and morbidity in this population. Fear of falling and imbalance contribute to physical inactivity, social isolation, loss of self confidence, anxiety, and reduced quality of life in older adults. Injuries resulting from falls account for two-thirds of unintentional injuries—one of the primary causes of death among the elderly. Moreover, half of the elderly individuals who are hospitalized due to fall-related injuries die within one year as a result of complications [56]. Therefore, assessing fall risk in elderly individuals is crucial for fall prevention.

Falls may result from intrinsic or extrinsic factors. Intrinsic risk factors include characteristics such as age, functional abilities, chronic diseases, and gait disorders [57], while extrinsic risk factors refer to conditions such as inappropriate footwear, slippery surfaces or loose rugs, tripping hazards, or poor lighting [58]. Identifying individuals at risk of falling is essential for developing fall prevention strategies and taking early measures. The simplest and most effective method for determining fall risk is to ask individuals whether they have a history of previous falls.

Several studies in the literature have aimed to predict fall risk using ML. Soylemez et al. [59] used the medical history, simple functional balance tests, and computerized dynamic posturography (CDP) data of elderly individuals (n = 120) in various ML models and developed 5 different models. Model 1 included individuals’ medical conditions and physical characteristics. Model 2 included functional balance tests. Model 3 included medical conditions, physical characteristics, and functional balance tests. Model 4 included CDP data. Model 5 incorporated all data. The authors reported the model accuracies as 87.5%, 83.34%, 100%, 91.66%, and 100%, respectively. They concluded that regardless of the reliability of balance tests used to predict fall risk, individuals’ medical conditions and physical characteristics must also be considered. Furthermore, they suggested that in clinics lacking objective tests such as CDP, fall risk can still be predicted with high accuracy by using medical conditions, physical characteristics, and functional balance tests in ML models. Although models 3 and 5 showed superior accuracy, their perfect performance raises concerns about potential overfitting, especially due to the limited sample size.

Fahimi et al. [60] applied 3 functional tests to elderly individuals and used the test data in ML models to predict fall risk, achieving the highest accuracy of 74.5% with the KNN algorithm. Ziegl et al. [61] developed an RF algorithm model using timed up and go test (TUG) data and reported that they could predict falls with a ROC-AUC of 96.9%. Sheng et al. [62] utilized questionnaire results, functional performance tests, and physical fitness tests from 215 elderly individuals across 7 ML algorithms, with model accuracies ranging from 0.933 to 0.950. In a retrospective study by Thapa et al. [63] using electronic health records, data from 2785 individuals were analyzed, and the extreme gradient boosting algorithm was identified as the best-performing model with a sensitivity of 84.8% and specificity of 70.6%.

In addition to accurately predicting fall risk, it is equally important for ML models to correctly identify individuals without fall risk. Therefore, evaluation metrics such as precision, sensitivity, specificity, and negative predictive value should be carefully considered, as misclassifying low-risk individuals may lead to unnecessary interventions and increased healthcare burden. Future ML studies should not only focus on fall risk prediction but also emphasize the accurate exclusion of low-risk populations to optimize clinical decision-making and resource allocation.

## 6. Clinical usability and limitations of machine learning

ML, although offering significant potential in the diagnosis and management of vestibular disorders, still has limited clinical use. Nowadays, some ML models have begun to be integrated into clinical processes through mobile applications and telemedicine platforms; however, most of these applications are still at the pilot stage, and there are various barriers to widespread clinical acceptance and implementation [64].

One of the foremost challenges involves regulatory and ethical concerns, particularly those related to patient data privacy and protection. Compliance with strict data regulations such as the General Data Protection Regulation (GDPR) in Europe, imposes technical and administrative burdens on the deployment of ML systems in healthcare environments. These regulations necessitate secure data handling, anonymization protocols, and explicit informed consent procedures that may not be readily compatible with all ML workflows, especially those requiring continuous data input or cloud-based processing.

Beyond legal considerations, several practical and infrastructural limitations also hinder clinical translation. For instance, the integration of ML tools into existing hospital information systems often requires significant technical adaptation and interoperability that many institutions—particularly those in resource-limited settings—are not equipped to implement. In addition, some ML applications depend on high-cost diagnostic devices (nystagmus analysis with ML) or wearable sensors that may not be readily available in standard clinical practice. Even when the necessary infrastructure is in place, a lack of training and familiarity among healthcare professionals can lead to skepticism, resistance to adoption, or improper use of the technology. The absence of standardized evaluation metrics and benchmarking datasets further complicates the validation and comparison of ML models across institutions, limiting generalizability and trust in real-world clinical settings. Taken together, these regulatory, technical, and human factors must be addressed through collaborative efforts involving clinicians, engineers, policymakers, and industry stakeholders to facilitate the responsible and effective integration of ML into vestibular healthcare.

## 7. Conclusion

The current literature indicates that ML algorithms have the potential to serve as effective and reliable tools in the diagnosis and prediction of vestibular disorders. ML facilitates the assessment of the complex symptomatology associated with vestibular dysfunction through objective data, thereby contributing to the development of clinical decision support systems. However, to enhance model performance and ensure generalizability, larger and multicenter datasets are needed. In this context, federated learning emerges as a promising solution by enabling model training on decentralized datasets across different centers while preserving patient privacy.

Although nystagmus analysis with ML offers valuable objectivity, its clinical implementation is limited by the requirement for specialized equipment and expertise, restricting its integration into telemedicine platforms. Home-based applications may be developed as an alternative; however, accurate recording of nystagmus in nonclinical environments remains technically challenging. Another feasible approach is the manual entry of clinical features and test results into secondary mobile applications that may enhance usability but also introduces the risk of clinician bias. Therefore, training ML models on multicenter datasets not only increases data diversity but also promotes standardization across clinical applications.

Moreover, current studies predominantly focus on common vestibular disorders such as BPPV, MD, and VN. Future research should also address rarer and more complex disorders, including vestibular paroxysmia, perilymph fistula, and central vestibular disorders. In such cases, the integration of radiological imaging techniques with ML algorithms may help identify these clinically ambiguous and frequently underdiagnosed conditions more effectively.

Finally, the applications of ML in the vestibular field extend beyond diagnosis and prediction. Future studies may utilize ML to forecast prognosis-such as the likelihood of developing residual dizziness-or to explore the psychosocial impacts of vestibular disorders, including effects on quality of life and depression.

In conclusion, the integration of ML-based systems into clinical practice has the potential to support early diagnosis of vestibular disorders and significantly contribute to the advancement of personalized treatment strategies.
